# Supplementary material for: The associations of dietary exposure to selected food additives with dietary patterns and overweight
Source: PLoS One. 2026 Feb 25;21(2):e0341825. doi: 10.1371/journal.pone.0341825 (PMC12935198; doi:10.1371/journal.pone.0341825)
Supplement: S1 Appendix — (DOCX) [file pone.0341825.s002.docx]

**Appendix 1.** Estimated daily intake of food additives (mg/kg/day) relative to the ADI.

| % respondents with a value higher than the ADI | % ADI | ADI (mg/kg/day) | Daily intake,  mean ± SD  (mg/kg/day) | Range of daily intake  (mg/kg/day) | Food additive  (mg/kg) |
| --- | --- | --- | --- | --- | --- |
|  | 4.56 | 25 | 1.14 ± 0.91 | 0.03-7.95 | E202 potassium sorbate |
| 0.5% | 8 | 0-0.07 | 0.0056 ± 0.014 | 0-0.21 | E250/E252 sodium/potassium nitrate |
|  |  | unlimited | 66.79 ± 76.46 | 0-769.51 | E282 potassium propionate |
|  | 1.69 | 75 | 1.27 ± 1.52 | 0-21.16 | E407 carrageenan (min) |
|  | 3.88 | 75 | 2.91 ± 3.51 | 0–43.56 | E407 carrageenan (max) |
|  | 2.71 | 70 | 1.90 ± 2.28 | 0–28.22 | E450_451_452  di/tri/poly phosphate |
|  | 0.38 | 15 | 0.0572 ± 0.19 | 0-2.49 | E955 sucralose |
|  | 3.36 | 9 | 0.3021 ± 0.88 | 0-8.73 | E950 acesulfame K |
|  | 0.22 | 7 | 0.0152 ± 0.052 | 0–0.69 | E952 cyclamic acid |
|  | 1.05 | 40 | 0.4182 ± 1.45 | 0–14.96 | E951 aspartame |

Mean ± SD: mean ± standard deviation.

Upon comparing the daily exposure per kilogram of body weight of the food additives, we found that the average exposure was below the acceptable daily intake. Only nitrite consumption was higher than the ADI, occurring in only 0.5% of the study participants. Comparable findings were also reported in studies conducted in other countries, where exposure to various food additives was examined (31, 32).
