# Supplementary material for: The associations of dietary exposure to selected food additives with dietary patterns and overweight
Source: PLoS One. 2026 Feb 25;21(2):e0341825. doi: 10.1371/journal.pone.0341825 (PMC12935198; doi:10.1371/journal.pone.0341825)
Supplement: S2 Appendix — (DOCX) [file pone.0341825.s003.docx]

**Appendix 2:**

**Association between weight status and dietary patterns:**

| P value (t test) | Overweight  BMI^1^: >25  N=302 (32.7%) | normal weight  BMI^1^:<=25  N=622 (67.3%) | Variables |
| --- | --- | --- | --- |
| 95% CI | M±SD | M±SD |  |
| 0.698  (56.03 - 37.86) | 460.63 ± 344.09 | 451.38 ± 337.73 | Ultra-processed energy (Kcal/day) |
| 0.084  (-82.52 – 5.15) | 353. 09 ± 340.01 | 314.41 ± 307.49 | Ultra-processed consumption weight (gram) |
| 0.441  (-0.009 – 0.21) | 0.21 ± 0.10 | 0.22 ± 0.11 | Ultra-processed energy proportion |
| 0.053  (-0.22 – 0.0001) | 0.09 ± 0.08 | 0.08 ± 0.07 | Ultra-processed weight proportion |
| <0.001  (0.798- 2.412) | 31.49 ± 5.82 | 33.09 ± 5.89 | Med55 score (12-50) |
| 0.115  (0.28 – 0.25) | 3.56 ± 0.97 | 3.67 ± 1.05 | Dash score (0.5-7.5) |
| 0.081  (-0.39- 0.669) | 9.84 ± 2.37 | 10.16 ± 2.67 | Sofi score (1-16) |

^1^ BMI. Body Mass Index

N±SD: mean ± standard deviation
