# Supplementary material for: The associations of dietary exposure to selected food additives with dietary patterns and overweight
Source: PLoS One. 2026 Feb 25;21(2):e0341825. doi: 10.1371/journal.pone.0341825 (PMC12935198; doi:10.1371/journal.pone.0341825)
Supplement: S3 Appendix — (DOCX) [file pone.0341825.s004.docx]

**Appendix 3:**

**Macro (gr/day) and micronutrients (mg/day) intake:**

| P value (t test) | Overweight  BMI^1^: >25  N=302 (32.7%) | normal weight  BMI^1^:<=25  N=622 (67.3%) | Macro nutrients |
| --- | --- | --- | --- |
|  | Daily intake  M±SD | Daily intake  M±SD |  |
| 0.414 | 224.45 ± 103.81 | 218.26 ± 109.99 | Carbohydrates (gr/day) |
| 0.096 | 106.96 ± 56.93 | 100.27 ± 57.36 | Total sugars (gr/day) |
| 0.004 | 95.65 ± 49.20 | 86.21 ± 40.66 | Protein (gr/day) |
| 0.007 | 84.19 ± 39.48 | 77.36 ± 34.13 | Total fat (gr/day) |
| 0.004 | 25.57 ± 14.51 | 22.82 ± 13.11 | Saturated fat (gr/day) |
| 0.011 | 34.15 ± 15.94 | 31.55 ± 13.75 | Monounsaturated (gr/day) |
| P value (t test) | Overweight  BMI^1^: >25  N=302 (32.7%) | normal weight  BMI^1^:<=25  N=622 (67.3%) | Micro nutrients |
|  | Daily intake  N±SD | Daily intake  N±SD |  |
| 0.185 | 19.10 ± 9.71 | 18.27 ± 8.57 | Poly saturated (gr/day) |
| 0.001 | 378.40 ± 305.25 | 314.16 ± 250.95 | Cholesterol (mg/day) |
| <0.001 | 0.17 ± 0.15 | 0.13 ± 0.12 | Trans fatty acids (mg/day) |
| 0.561 | 32.60 ± 18.08 | 31.89 ±17.19 | Total fiber (gr/day) |
| 0.055 | 1141.43 ± 714.84 | 1052.55 ± 630.42 | Calcium (mg/day) |
| 0.214 | 13.37 ± 6.96 | 12.84 ± 5.77 | Iron (mg/day) |
| 0.302 | 540. 30 ± 244.90 | 523.77 ± 219.83 | Magnesium (mg/day) |
| 0.109 | 2. 71 ± 1.31 | 2.57 ± 1.19 | Vitamin B6 (mg/day) |
| 0.009 | 5.90 ± 6.44 | 4.88 ± 3.26 | Vitamin B12 (mg/day) |
| 0.158 | 462.40 ± 274.12 | 438.81 ± 218.79 | Folate (mg/day) |
| 0.183 | 7.43 ± 6.02 | 6.89 ± 5.60 | Vitamin D (mg/day) |
| 0.001 | 3803.51 ± 1911.44 | 3392.55 ± 1480.09 | Sodium (mg/day) |
| 0.049 | 2106.97 ± 931.34 | 1985.70 ± 852.26 | Total Energy (Kcal/day) |

^1^ BMI. Body Mass Index

M±SD: mean ± standard deviation

We observed a statistically significant higher intake (p–value < 0.05) of certain macronutrients, including total sugars, protein, total fat, saturated fat, monounsaturated fat, cholesterol, and trans fatty acids, among respondents who are overweight. Additionally, we found that overweight respondents had a higher consumption of certain micronutrients, such as calcium, vitamin B12, and sodium. Furthermore, the total daily caloric intake was significantly higher in respondents who are overweight.

**Macronutrient intake (proportion of total calories) according to weight status**

| P value (t test) | Overweight  BMI^1^: >25  N=302 (32.7%) | normal weight  BMI^1^:<=25  N=622 (67.3%) | Calories of macronutrients (proportion) |
| --- | --- | --- | --- |
|  | % of calories  M±SD | % of calories  M±SD |  |
| 0.047 | 44.15 ± 7.51 | 45.23 ± 7.92 | Calorie proportion carbohydrates |
| 0.055 | 18.74 ± 3.91 | 18.18 ± 4.17 | Calorie proportion protein |
| 0.162 | 37.12 ± 5.22 | 36.58 ± 5.56 | Calorie proportion Total fat |
| 0.014 | 11.12 ± 2.97 | 10.57 ± 3.28 | Calorie proportion Saturated fat |

^1^ BMI. Body Mass Index

Using t-test

M±SD: mean ± standard deviation

When analyzing the distribution of calories derived from macronutrients, it was discovered that individuals of normal weight have a higher percentage of calories coming from carbohydrates compared to those who are overweight (45.23% and 44.15% respectively, p-value = 0.047). Conversely, individuals who are overweight have a higher percentage of calories coming from saturated fat, as compared to those of normal weight (11.12% and 10.57% respectively, p-value =0.014).
